# Novel prognostic tools that identify high-risk follicular lymphoma

**DOI:** 10.1097/HS9.0000000000000214

**Published:** 2019-06-30

**Authors:** Stefano Luminari

**Affiliations:** 1 Hematology Unit, Azienda Unità Sanitaria Locale—IRCCS, Reggio Emilia, Italy; 2 Surgical, Medical and Dental Department of Morphological Sciences related to Transplant, Oncology and Regenerative Medicine, University of Modena and Reggio Emilia, Reggio Emilia, Italy

**Take home messages**

- In 20% to 30% of patients with follicular lymphoma (FL), the disease shows an aggressive behavior.
- Novel biomarkers are available in FL each with a different ability to identify high-risk patients.
- Further improvement in the management of FL will likely be achieved by means of risk adapted therapies.

## Introduction

For many years, risk in follicular lymphoma (FL) has been defined with conventional clinical prognostic factors and indexes with the follicular lymphoma international prognostic indexes (FLIPI and FLIPI2) being the most frequently used scores.^1^,^2^ None of these indexes, however, has ever been able to unequivocally identify high-risk patients.

## Current state of the art

Recently, Casulo et al^3▪^ correlated the concept of high-risk FL with time to progression. The authors showed that patients with high tumor burden FL who progress or relapse within 24 months (POD24) after immunochemotherapy (here: Cyclophosphamide, Doxorubicin, Vincristine, Prednisone [CHOP] with the anti-CD20 antibody Rituximab [R]) had a significantly shorter overall survival (OS) compared with patients without POD24. These findings were recently validated in independent FL patient cohorts and with immunochemotherapy regimens different from R-CHOP.^4^,^5^

POD24 is an important step toward a better understanding of FL; however, patients would rather benefit from a better risk stratification closer to FL diagnosis, thereby allowing the development of risk-modifying approaches. In that respect, the heterogeneity of high-risk FL which is so far defined by refractoriness and transformation needs to be better understood. These patients’ higher risk of dying is mainly caused by lymphoma^6^ and might be driven not only by a more aggressive biology of FL but also by refractoriness to immunochemotherapy and by a higher risk of transformation. Indeed, the combination of different dimensions contributes to increasing the risk in FL. In this context, novel tools have recently been studied to identify high-risk FL, with most of the available data coming from the analysis of molecular, pathologic, and metabolic features of the disease.

### Baseline biomarkers

A number of studies have found associations between several pathologic features such as histologic grading, proliferation index, and microenvironment in diagnostic FL biopsies and varying degrees of disease aggressiveness, but have not confirmed these features as reliable prognosticators in the era of immunochemotherapy.^7^ Advanced noninvasive methods for the detection of cell-free DNA in general and more specifically of circulating tumor DNA are underway, to determine the tumor load which could be used for pretherapeutic risk assessment.^8^

Two attempts have been made to integrate clinical prognostic factors with molecular biomarkers: Pastore et al^9▪^ integrated the mutational status of 7 genes recurrently mutated in FL in the context of the FLIPI backbone and Huet et al^10^ used gene expression analysis to identify a 23-gene predictor model. Both the m7-FLIPI and the 23-gene model identified a high-risk group of 28% and of 21% to 35% of patients, respectively, who had a shorter PFS. A simplified version of m7-FLIPI was also validated allowing to predict the risk of POD24 in up to 80% of high-risk patients.^11^

Finally, since ^18^F-fluordesoxyglucose (FDG) avidity was confirmed in the majority of FL, the prognostic value of quantitative parameters obtained from baseline FDG-PET/computed tomography has been analyzed. Of these parameters, standardized uptake value (SUV) has been shown to be a good tool to identify areas at higher risk of histologic transformation and could thus be used to guide diagnostic biopsies. More importantly, in a recent study by Meignan et al,^12^ baseline total metabolic tumor volume (TMTV), defined as the sum of the volumes of sites with an SUV value above a significant threshold, has been confirmed as the strongest pretreatment prognostic factor, able to identify a third of patients at higher risk of progression and of dying from FL, independently of FLIPI and FLIPI2 (Table ^1^).

**Table 1:**
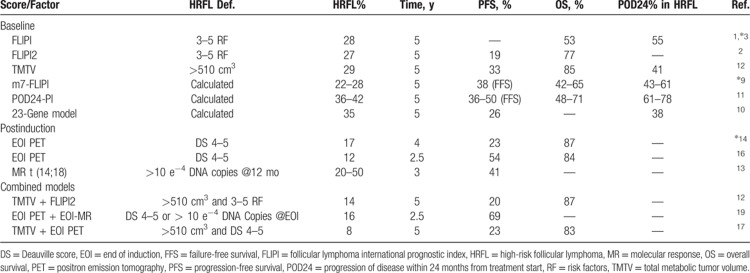
Summary of Prognostic Factors Used to Identify HRFL Patients and Correlation With POD24

The above-mentioned molecular and metabolic biomarkers represent new tools to identify high-risk patients at diagnosis and might be used to support biology guided therapies (ie, EZH2 inhibitors). However, they both show limitations in their reproducibility and require further investigations in the context of prospective studies and in different subgroups of FL patients (ie, low tumor burden cases and patients treated with new drugs).

### Postinduction prognostic tools

Response to therapy assessed either with FDG-PET or with highly sensitive molecular techniques that are able to measure cell-free DNA or to determine low levels of the t(14;18) chromosomal translocation (minimal residual disease [MRD]) have recently been suggested as useful prognostic tools.^13^–^15^ Trotman et al recently reported the results of the largest study ever conducted, to investigate the prognostic role of metabolic response in more than 500 patients with treatment-naïve advanced-stage FL enrolled in the GALLIUM trial. The authors were able to confirm that metabolic response to induction immunochemotherapy is prognostic both for PFS and OS, and that Lugano response criteria are accurate and reproducible in FL. More importantly, this study showed that metabolic response is associated with prognosis in nearly all advanced-stage FL patients, including those who receiving maintenance therapy and those who treated with the new generation anti-CD20 monoclonal antibody (ie, obinutuzumab) and different chemotherapy backbones.^16^

## Future perspective

In summary, several biomarkers and prognostic factors are currently available to identify a subgroup of approximately 20% to 30% of patients with FL whose lymphoma show an aggressive clinical behavior. The use of novel techniques to measure cell-free or tumor-free DNA holds promises to a deeper understanding of FL heterogeneity, and for a better monitoring of response to treatment, hopefully leading to the identification of novel biomarkers.^8^ Each available biomarker has a different ability to predict outcome and likely describes different features of the higher individual risk. Since none of the prognostic factors identified so far is currently available to accurately identify high-risk FL and applies to the clinical and biological heterogeneity of FL, a reasonable strategy might be to combine available factors. Indeed, recent results showed that baseline and postinduction factors can be successfully combined (ie, TMTV + FLIPI2, TMTV + metabolic response, metabolic response + molecular response).^12^^,^^17^^,^^18▪^^,^^19^,^20^ Clinical trials are underway that investigate the efficacy of a response-adapted approach, based on the use of novel prognostic biomarkers including FDG-PET and/or MRD, aiming to tailor the postinduction maintenance phase of therapy to the quality of response (NCT02063685 and EudraCT 2016-004010-10).
